# Supplementary material for: Size- and Chirality-Dependent Structural and Mechanical Properties of Single-Walled Phenine Nanotubes
Source: Materials (Basel). 2023 Jun 29;16(13):4706. doi: 10.3390/ma16134706 (PMC10342797; doi:10.3390/ma16134706)
Supplement: Supplementary file 1 [file materials-16-04706-s001.zip › materials-2429746-supplementary.pdf]

Size- and Chirality-Dependent Mechanical Properties of Single-Walled Phenine Nanotubes

Yanjun Liu[1], Ruijie Wang[1]*, Liya Wang[1], Jun Xia[1], Chengyuan Wang[2] and Chun Tang[1,3]*

1 Faculty of Civil Engineering and Mechanics, Jiangsu University, Zhenjiang 212013, China

2 Zienkiewicz Centre for Computational Engineering, Faculty of Science and Engineering, Swansea University, Bay Campus, Swansea, Wales SA1 8EN, UK

3 Key Laboratory for Intelligent Nano Materials and Devices of Ministry of Education, , Nanjing University of Aeronautics and Astronautics, Nanjing 210016, China

*e-mail:wangruijie@ujs.edu.cn (R. W.), tangchun@ujs.edu.cn (C. T.)

Supporting Materials

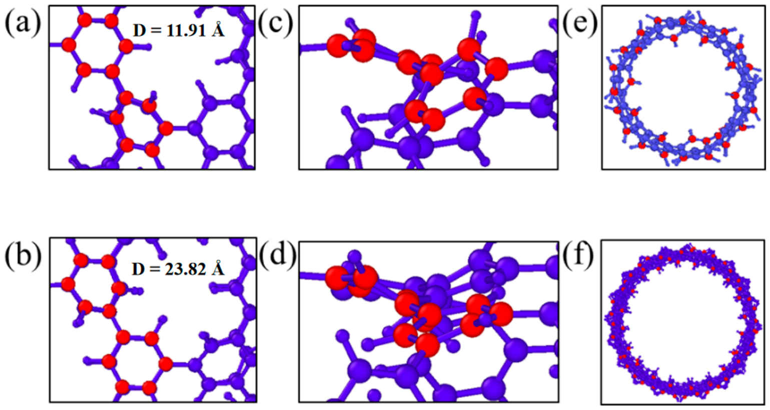

Figure S1.Twisting behavior of linear carbon chains in PNTs. a and b, local structures of twisted benzene rings during relaxation, c and d, side view of the adjacent benzene ring after twisting, e and f, cross-section of the corresponding PNTs. The atoms marked in red are carbon atoms in the benzene ring structure that undergo torsion.

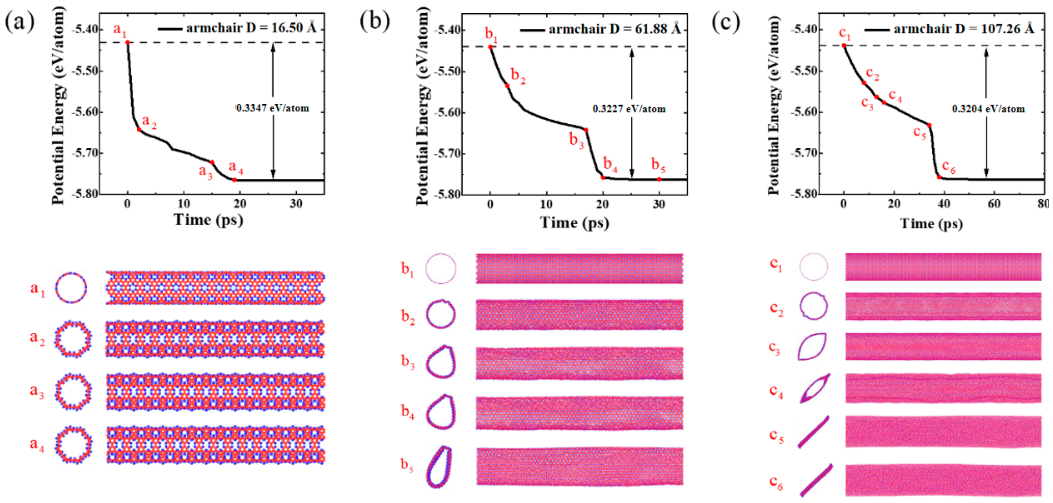

Figure S2. Structural evolution for 3 selected armchair PNTs as a function of time, the corresponding potential energy are plotted in the top panel, while the snapshots are shown in the bottom.

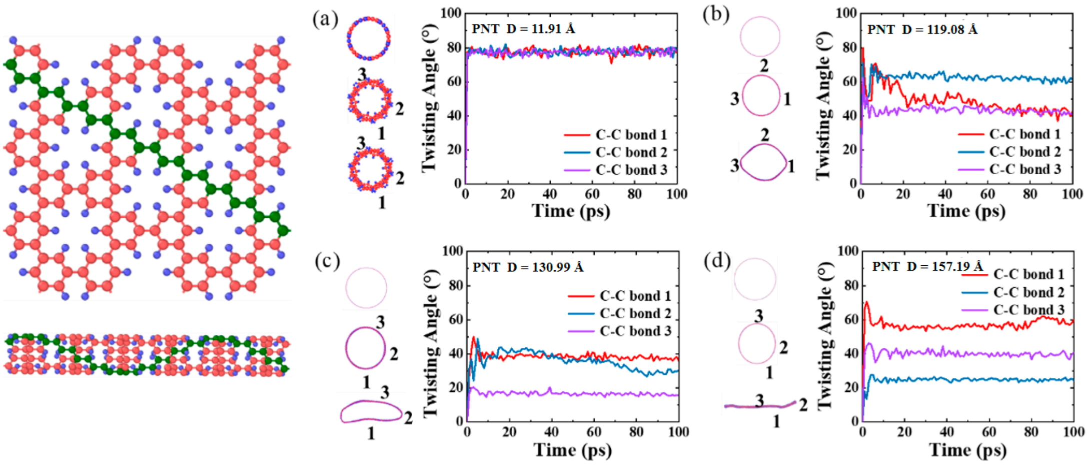

Figure S3. Twisting of helical carbon chains in zigzag PNTs. (a-d) present selected twisting angle evolution as a function of time for PNTs with different diameters.

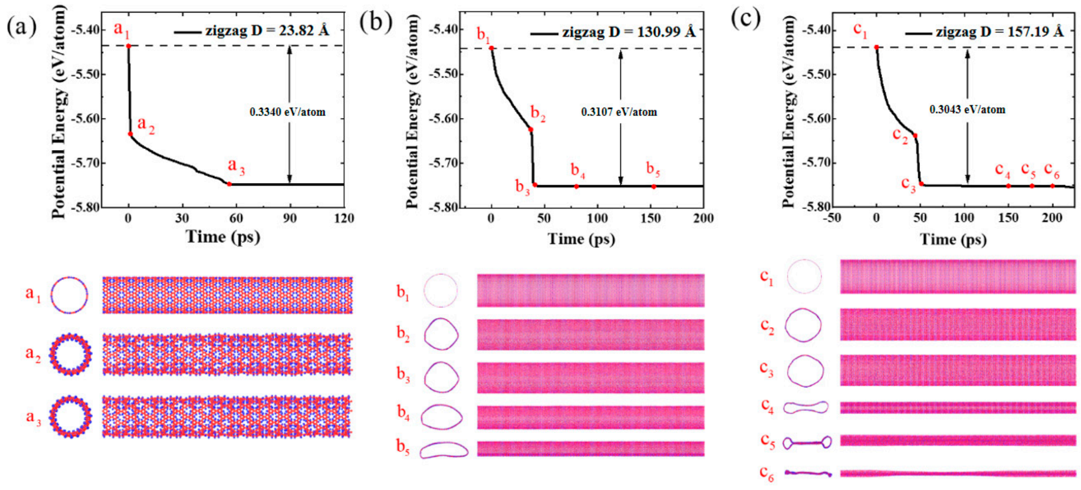

Figure S4. Structural evolution for 3 selected zigzag PNTs as a function of time, the corresponding potential energy are plotted in the top panel, while the snapshots are shown in the bottom.

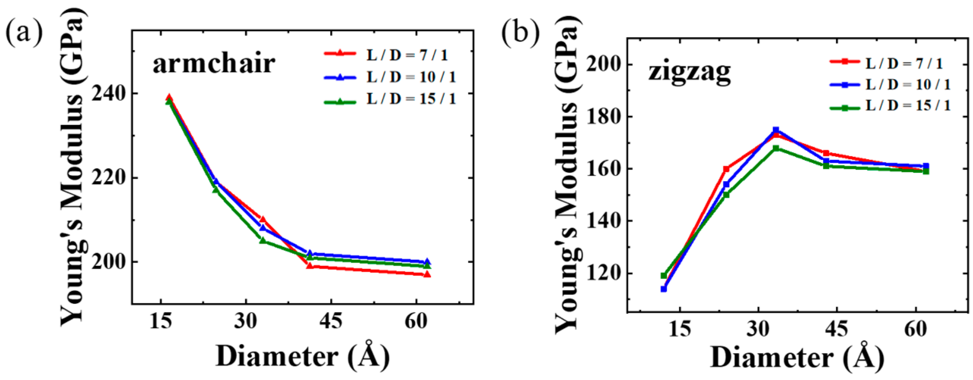

Figure S5, Young's modulus of PNTs under tensile loadings.

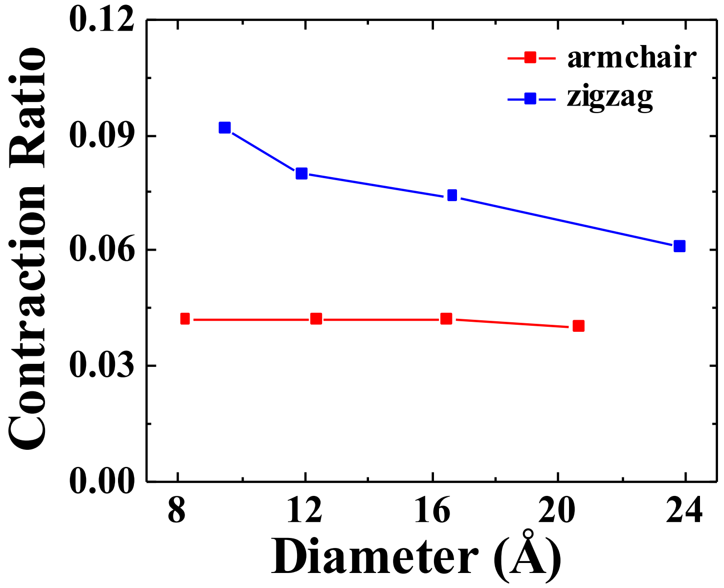

Figure S6, Contract of PNT length when rolled up from defected graphene sheets.
